# Static and Dynamic Motor Control in Active Young Adults: Associations with Oswestry Disability Index and Functional Movement Screen Asymmetries

**DOI:** 10.3390/healthcare14091223

**Published:** 2026-05-02

**Authors:** Julio Martín-Ruiz, Iván Chulvi-Medrano

**Affiliations:** 1 Department of Health and Functional Assessment, Catholic University of Valencia, 46900 Valencia, Spain; 2 Research Group in Prevention and Health in Exercise and Sport (PHES), Department of Physical Education and Sports, University of Valencia, 46022 Valencia, Spain; ivan.chulvi@uv.es

**Keywords:** low back pain, physical activity, motor control, functional assessment

## Abstract

**Highlights:**

**What are the main findings?**

- Greater trunk endurance on the McGill tests was associated with lower disability in 71 physically active university students with minimal lumbar disability (Oswestry Disability Index 4.00 ± 5.69).
- Dynamic screening added distinct information: total Functional Movement Screen showed only a weak, non-significant inverse relationship with Oswestry Disability Index (β = −0.83, *p* = 0.071), but the low-prevalence Inline Lunge asymmetry (5.6%) was exploratorily associated with a higher Oswestry Disability Index (*p* = 0.011; FDR = 0.042), unlike Rotary Stability asymmetry.

**What are the implications of the main findings?**

- In physically active young adults with very low disability levels, trunk endurance tests and selected movement-pattern measures provided complementary descriptive information on current lumbar-related function. However, the observed associations with ODI were modest, the explanatory capacity of the multivariable model was low, and the finding related to Inline Lunge asymmetry should be interpreted as exploratory because of the very small subgroup size. Accordingly, these results should be understood as population-specific and hypothesis-generating rather than predictive or directly clinically applicable. Their main contribution is to describe concurrent associations in a low-disability active cohort and to help guide future studies in broader and more clinically relevant populations.
- When disability levels are low, the total Functional Movement Screen score may have limited explanatory value, and specific asymmetries should be interpreted cautiously and as exploratory findings, particularly when subgroup frequencies are very small.

**Abstract:**

Background: Low back pain (LBP) is a leading cause of disability, particularly in young adults. Decreased trunk endurance and altered movement patterns have been associated with lumbar symptoms and functional limitations; however, their concurrent relationships in active populations with minimal disability remain insufficiently characterized. This study was designed as an exploratory cross-sectional analytical study. Methods: The sample comprised 71 physically active university students (mean age, ~23 years; 79% men). Trunk endurance was assessed using the McGill isometric tests, and selected movement-pattern measures were obtained from four Functional Movement Screen (FMS) tasks focused on lumbopelvic control. The total FMS score was calculated, asymmetries were recorded in the Inline Lunge and Rotary Stability tasks, and lumbar-related disability was measured using the Oswestry Disability Index (ODI). Associations were analyzed using correlations and adjusted linear regression, and asymmetry-based comparisons were evaluated using non-parametric tests. Results: The average ODI was very low (approximately 4%), suggesting a floor effect. Greater trunk endurance was associated with lower ODI values, whereas the association between total FMS and ODI was weak and did not reach statistical significance in the adjusted model. Inline Lunge asymmetry was associated with higher ODI values, but this finding should be interpreted cautiously because of the very small subgroup size. Conclusions: In this physically active young adult sample, trunk endurance and selected movement-pattern measures provided complementary descriptive information on lumbar-related function; however, the observed associations were modest and should be interpreted cautiously.

## 1. Introduction

Low back pain (LBP) is one of the top 10 causes of disability worldwide [1] with a lifetime prevalence of 60–80% in the general population [2,3], including 84% of university students [4,5] due to causes such as stress or poor posture.

Proper conditioning of the intrinsic trunk muscles is essential for adequate static and dynamic stability [6] and improves stiffness, which indicates greater resistance to deformation [7], an aspect that is clearly diminished in individuals with LBP [8,9]. It has been observed in young people, in whom a shorter time maintaining the plank (Sorensen) was associated with greater LBP and worse Oswestry Disability Index (ODI) scores [10,11]. Therefore, deficient static motor control contributes to spinal limitations and dysfunction. Dynamic functional tests may better reflect movement performance in everyday tasks [12,13], and the Functional Movement Screen (FMS) is commonly used to assess movement patterns [14]. Previous studies have reported lower FMS scores in individuals with LBP than in healthy controls [15], although the predictive validity and clinical utility of the total FMS score remain debated, particularly in low-risk and physically active populations [16,17]. Therefore, individual patterns and asymmetries may be more informative than composite scores alone. ODI was selected because it is a widely used instrument for lumbar-related disability; however, in minimally symptomatic and physically active populations, its low score range may reduce sensitivity for detecting subtle functional differences. Accordingly, its interpretation in the present sample should be considered cautious and contextual.

Both elements (static and dynamic control) are complementary; therefore, an integrated approach would be ideal, although the interaction between them has not yet been clarified [14,18]. Most studies have focused on the isolated analysis of a single variable; therefore, more specific research on active populations and athletes is required [8]. The literature on LBP in university students is limited and heterogeneous; the available reviews include few studies and lack incidence data, as is the case with medical students [19]. Furthermore, research on prevention in students is insufficient to draw firm conclusions, owing to the scarcity of high-quality studies [20]. This shortage is also observed among university students in the health field, where evidence on the epidemiology and associated factors of LBP is recognized as very limited [21]. Finally, studies that incorporate objective physical tests—both static and dynamic—are uncommon; when they do appear, they typically combine static posture and functional kinematics without providing a sufficient basis for evidence-based prevention, or treatment protocols [22].

Therefore, this study aimed to examine the association between static motor control assessed with the McGill tests and dynamic motor control assessed with selected Functional Movement Screen tasks, and their relationship with lumbar pain/disability measured with the Oswestry Disability Index in physically active young adults. The objective was to provide an exploratory, population-specific analysis of concurrent associations in a homogeneous active sample, without implying prediction or broad clinical generalizability. We hypothesized that trunk endurance tests and selected movement-pattern measures would show modest associations with ODI and provide complementary descriptive information within this specific cohort.

## 2. Materials and Methods

### 2.1. Study Design and Ethical Considerations

This was an exploratory cross-sectional analytical study conducted in physically active university students. The study was performed in accordance with the Declaration of Helsinki and was approved by the Ethics Committee of the Catholic University of Valencia (protocol code UCV/2025-26/002). All participants provided written informed consent before inclusion.

### 2.2. Participants

The participants were active university students in sports science aged 22–26 years. Individuals who practiced physical activity for four or more days per week and regularly attended classes were included. Participants with LBP or those who had recovered from an injury less than two months prior to the measurement were excluded. Participation was entirely voluntary, with no academic consequences for non-participation. Identifying information was collected only to link questionnaire responses with study IDs during data processing and was not used in the analyses.

### 2.3. Procedure

All assessments were performed by a single examiner following the same procedure for all participants. The testing sequence was kept consistent to reduce procedural variability; however, no formal intra-rater or inter-rater reliability study was conducted in this sample. In addition, although participants with recent musculoskeletal problems were excluded, the fixed recovery window was chosen pragmatically to minimize interference from unresolved symptoms and should be interpreted as an operational eligibility criterion rather than a universal recovery threshold.

All participants completed a specific questionnaire on LBP and underwent an isometric spinal stability test and four functional tests. The association between static and dynamic motor control tests was examined, along with their relationship with LBP-related disability. A single session was conducted during university class hours and voluntarily, beginning with a standardized warm-up, in November 2025 at the Faculty of Physical Activity and Sports Sciences.

At the beginning of the study, weight (Seca 750, Hamburg, Germany), height (Seca 213, Hamburg, Germany), and biacromial distance were measured for each participant. The participants were given 15 min to complete the Oswestry Disability Index (ODI) questionnaire, a specific resource for people with lower back pain that assesses the extent to which this problem affects functional capacity and daily activities. It is used to measure the degree of disability and to determine whether the patient improves or worsens with treatment [23]. Participants were asked to complete all ODI items. To allow linkage between questionnaire responses and study records, each participant was assigned a study ID during data processing; identifying information, when provided for administrative linkage, was stored separately and was not used in the analyses.

The next test was the McGill lumbar stability test [24], an isometric exercise protocol designed to determine the endurance and core stability of the trunk muscles, in which the participant must hold the position for as long as possible. First, spinal extension was performed while the participant was in the prone position. The participant was positioned prone on a stretcher, lying with their hips at the edge, and using the anterior superior iliac spine as the last point of support. The legs were secured with two straps, one under the gluteus and the other over the gastrocnemius muscles. The hands were crossed and placed on the opposite shoulder, and the timer was activated to maintain the isometric extension of the spine. Next, bilateral trunk flexion was conducted. From a seated position, both knees were bent, arms crossed with each hand touching the opposite shoulder, and the back rested on a 60° surface. After 10 s, the supports were removed. The final test was a side plank on both sides. From a lateral lying position without support from the legs or knees, the lateral abdominal muscles were contracted. In all attempts, the position was held for as long as possible, stopping when fatigue and posture alterations became evident. The recovery interval between exercises was 2 min to avoid fatigue. The test indices used the extension of the spine as a reference (100%). Normal trunk flexion is 99% in men and 80% in women. For the side planks, the values were 64% and 40% (men and women) on the right and 62% and 38% on the left, respectively.

Finally, four FMS tests were conducted [17], those with greater involvement of the stabilizing muscles of the spine: (1) Deep Squat: with the participant standing, feet shoulder-width apart, and holding a bar overhead, they performed a Deep Squat. (2) Inline Lunge: standing with feet on a line separated by the length of the tibia, holding a bar with both hands behind the back in a vertical position, the participant performed knee flexion-extension until the rear knee touched the ground. This was performed on both sides of the body. (3) Rotary Stability: in the quadruped position, the participant extended the hip and knee while flexing the shoulder and extending the elbow on the same side of the body until both the leg and arm were parallel to the ground, marking the position. This exercise was bilateral, similar to the previous exercise. (4) Trunk Stability Push-Up: from a prone position, with the elbows bent and hands resting on the ground, the participant performed a push-up in one movement, simultaneously lifting the shoulders, hips, and knees.

All McGill and FMS assessments were performed by a single examiner following standardized testing procedures. No formal intra- or inter-rater reliability analysis was conducted in the present sample.

**Figure 1:**
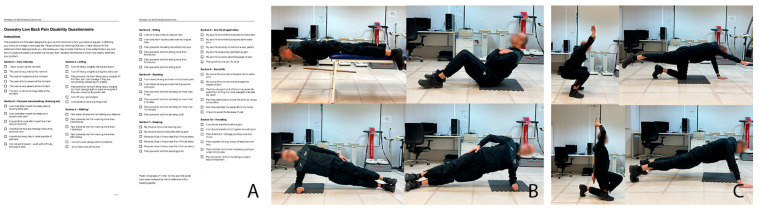
(**A**), Oswestry Disability Index; (**B**), McGill exercises; (**C**), FMS test.

Each exercise was performed one to three times. If the task was performed correctly on the first attempt, no further attempts were made; otherwise, there were two possibilities. The score was recorded as 0 when it was not possible to perform, 1 when performed with pain, 2 when completed with compensation, and 3 when performed correctly, with a maximum score of 12. A 30 s pause was taken between each attempt (Figure 1).

### 2.4. Statistical Analysis

The analysis was performed using R (R Core Team, v.4.5.2., Viena, Austria) [25] with openxlsx (v. 4.8.2.1.) [26] for the tables and ggpubr (v. 0.6.3.) [27] for the figures. An exploratory analytical approach with nonparametric methods was used when appropriate, given the non-normal behavior of clinical variables and the imbalance between groups in some comparisons (e.g., low frequency of asymmetries in certain FMS items). Prior to the analyses, the database was cleaned by verifying logical ranges, recoding categorical variables, and assessing missing values. Continuous variables were described using the mean and standard deviation, median and interquartile range, and minimum and maximum values. Categorical variables were summarized using absolute frequencies and percentages.

Given the study’s objective, static motor control was operationalized using the McGill tests (endurance times in seconds and derived indices), and dynamic motor control was measured using the Functional Movement Screen, both in terms of the total score and selected items, and asymmetry indicators. Disability associated with LBP was quantified using the Oswestry Disability Index as the primary clinical outcome.

The Wilcoxon rank-sum test (Mann–Whitney U) was used to compare continuous variables between two independent groups defined by sex or the presence/absence of asymmetry (e.g., Inline Lunge asymmetry or Rotary Stability asymmetry); the test statistic and *p*-value are reported. This procedure was considered appropriate because of its robustness to non-normal distributions and unequal sample sizes. In the analysis of the associations between the continuous variables of static and dynamic motor control and disability, Spearman’s correlation coefficients (*ρ*) with their respective *p*-values were estimated, and the structure of the associations was represented using a correlation heatmap.

Because non-parametric methods were used for group comparisons, the corresponding tables were revised to improve consistency of descriptive reporting and interpretation, and age was retained as a relevant adjustment variable because of the observed sex-related difference.

When multiple simultaneous contrasts were performed within the same family of hypotheses (for example, several outcome comparisons against signs of asymmetry or large correlation matrices), the error due to multiplicity was controlled by applying a false discovery rate (FDR) correction using the Benjamini–Hochberg procedure, with both raw *p*-values and FDR-adjusted *p*-values reported. Contrasts were considered statistically significant at a two-tailed threshold of α = 0.05; however, findings from low-frequency subgroups were interpreted cautiously and as exploratory.

Finally, to explore the joint contribution of dynamic motor control and covariates to disability, linear regression models (ordinary least squares) were fitted with the total ODI as the dependent variable and total FMS and potential confounding covariates (age, body mass index, and sex) as predictors. The β coefficients, standard errors, test statistics, and *p*-values are reported, as well as the global model fit metrics (e.g., R and adjusted R).

Sex-specific reference ratios were not included in the primary models because the aim was to examine associations based on the observed continuous values within the study sample. Instead, sex was entered as a covariate in the multivariate regression model. The McGill sum of indices was included as a complementary descriptive variable. Because universally accepted clinical cut-offs are lacking and normative reference values are context-dependent, they were not used as standardized primary predictors in the main analyses.

## 3. Results

A total of 71 participants were included in the analysis. The sex distribution was 56 men (78.9%) and 15 women (21.1%). Regarding lumbar disability, the total ODI showed low values, with a mean of 4.00 (± 5.69). The complete descriptive statistics for the continuous and categorical variables are presented in Table 1.

Asymmetry in the Inline Lunge was uncommon and was observed in 5.6% of participants. Rotary Stability was observed in 15.5% of the participants.

When comparing the results by sex using non-parametric tests (Wilcoxon rank-sum test), a significant difference was observed in age (*p* = 0.004). In contrast, no differences were found according to sex in the total ODI (*p* = 0.776), total FMS (*p* = 0.096), or BMI (*p* = 0.228). Taken together, this suggests that in this sample, lumbar disability and functional performance, as assessed by the FMS, were comparable between sexes, although age differed (Table 2).

When comparing asymmetry, Inline Lunge asymmetry showed a difference in total ODI (raw *p* = 0.011; FDR-adjusted *p* = 0.042); however, this finding should be interpreted cautiously because the asymmetry subgroup was very small (*n* = 4). In contrast, the association with total FMS score did not remain significant after FDR correction (raw *p* = 0.052; FDR-adjusted *p* = 0.103) (Table 3).

In contrast, asymmetry in FMS Rotary Stability showed no association with the total ODI score (*p* = 0.805, adjusted *p* = 0.808) or total FMS score (*p* = 0.808, adjusted *p* = 0.808).

Associations between continuous variables were evaluated using Spearman’s correlation and false discovery rate (FDR) adjustment. High correlations were observed among the components of the McGill endurance test, notably the relationship between the McGill side bridge left and McGill side bridge right with *ρ* = 0.897 (FDR-adjusted *p* ≈ 0), which is consistent with high concordance in lateral performance.

**Figure 2:**
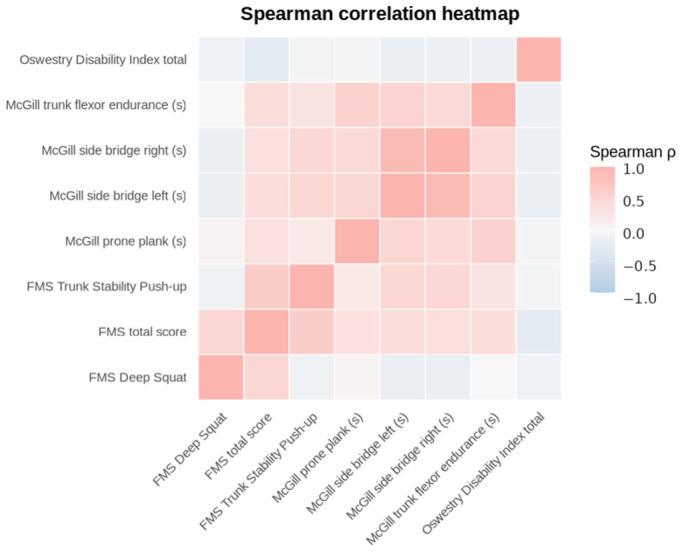
Correlation heatmap (Spearman’s ρ).

Within FMS, Trunk Stability Push-up showed a moderate correlation with the total FMS score (*ρ* = 0.626, FDR-adjusted *p* = 0.001), and Deep Squat was also associated with the total score (*ρ* = 0.480, FDR-adjusted *p* = 0.001), supporting that these items make a relevant contribution to overall FMS performance. The complete correlation matrix is presented in Figure 2, and the main associations are presented in Table 4.

An ordinary least squares (OLS) linear regression model was fitted to explain the total ODI as a function of the total FMS, stratified by age, BMI, and sex. The coefficient for total FMS was negative, indicating that greater functional performance was associated with less disability, although this did not reach statistical significance (β = −0.83, *p* = 0.071) (Table 5).

**Figure 3:**
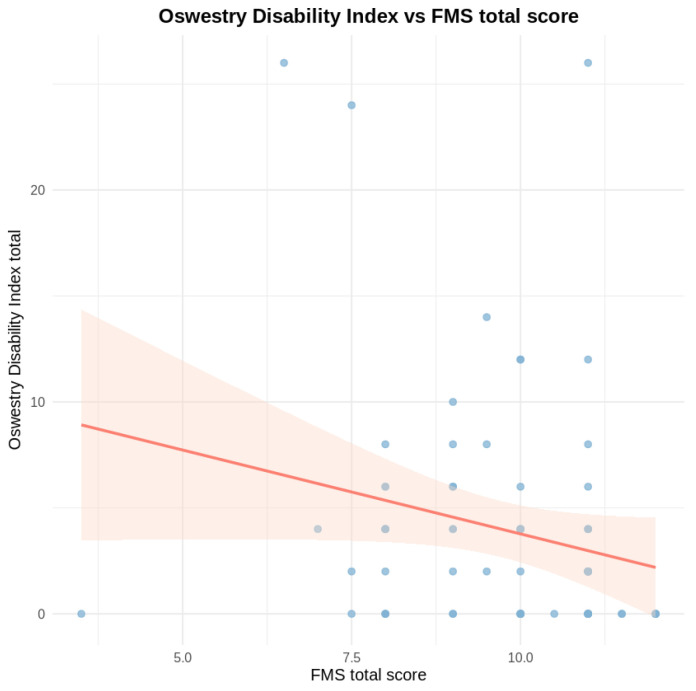
Relationship between Oswestry Disability Index and Functional Movement Screen total score. Note: Each blue point represents an individual participant. The red line shows the fitted linear regression line, illustrating the overall tendency for ODI scores to decrease as FMS total scores increase. The shaded area represents the 95% confidence interval around the regression line. The wide dispersion of points indicates substantial inter-individual variability and supports a cautious interpretation of this association.

The model showed limited explanatory capacity (R^2^ = 0.059), indicating that the total ODI in this cohort depended on other factors not included in this analysis (such as training load, pain history, sports experience, and psychosocial variables). The bivariate relationship between total ODI and total FMS scores is presented in Figure 3.

## 4. Discussion

Trunk muscle endurance is associated with the degree of disability, with an inversely proportional relationship, showing functional deterioration, in accordance with previous studies [10]. Our findings reinforce that poor performance in the McGill test supports an association between poorer trunk endurance and greater LBP-related disability [28]. Therefore, the interpretation of the McGill sum of indices should be considered descriptive, and reference ratios can be considered supportive interpretive tools rather than definitive diagnostic thresholds. In the dynamic aspect, it was also confirmed that poorer dynamic control corresponded to a higher ODI [29], an element supported by other studies that indicated a significant negative correlation between FMS scores and ODI in individuals with chronic LBP [14]. The present study provides these observations in active young individuals, highlighting that selected FMS outcomes may provide complementary descriptive information on lumbar-related function in this population.

Not all studies agree on the predictive value of FMS tests; recent research has found no significant differences in total FMS scores between athletes with and without back pain [4]. Therefore, functional improvement does not eliminate pain. Other factors, such as individual habits and body mass index (BMI), may also determine LBP. Therefore, LBP is multifactorial [2,30], and the interpretation of this study should be applied in the context of individual characteristics. Overall, integrating measures of static and dynamic motor control with a clinical index of pain/disability may help provide a broader descriptive profile of function in young participants [31,32].

From a research perspective, combining McGill and Functional Movement Screen measures with ODI may help describe different dimensions of current functional status in physically active young adults. However, given the cross-sectional design, the very low ODI values, and the limited explanatory capacity of the regression model (R^2^ = 0.059), these measures should not be interpreted as tools for risk prediction or direct clinical decision-making in this population. At most, the present findings support further exploratory research in broader and more heterogeneous samples. Although exercise-based approaches such as core-focused training and movement re-education have shown benefits for pain and disability in clinical low back pain populations [33], those findings come from different study contexts and should not be directly extrapolated from the present dataset. Accordingly, any practical implications for exercise or rehabilitation derived from the current results should be considered indirect and hypothesis-generating rather than directly supported by this study.

Considering this evidence, in young adults with low overall disability, both trunk endurance tests and selected movement-pattern measures provided complementary descriptive information; however, their associations with ODI were limited. In the multivariable model, a higher total FMS score was associated with a lower ODI score, but this relationship did not reach statistical significance, and the overall explanatory capacity of the model was low (R^2^ = 0.059). Inline Lunge asymmetry showed a difference in ODI after multiplicity adjustment; however, because this subgroup was very small, this observation should be interpreted cautiously as an exploratory finding. Rotary Stability asymmetry was not associated with ODI or total FMS score. Overall, the very low ODI values observed in this sample suggest a floor effect that likely reduced variability and limited the ability to detect stronger associations.

### Limitations

This study was cross-sectional; therefore, the associations observed should be interpreted as concurrent relationships without causal inference. Several limitations should be considered. First, the sample showed very low ODI values, which likely produced a floor effect, restricted variability, and reduced sensitivity for detecting stronger or clinically interpretable associations. Second, the cohort was homogeneous and composed of physically active young adults, which limits external validity; therefore, the findings should be regarded as population-specific and should not be extrapolated to clinical or more heterogeneous populations. Third, some subgroup analyses were based on low-prevalence asymmetries, particularly Inline Lunge asymmetry (5.6%), which reduced statistical precision and supports interpreting these findings as exploratory. Fourth, ODI, although widely used, may have limited sensitivity in minimally symptomatic active populations and therefore may have constrained the detection of subtle functional differences. Nevertheless, it was retained because it remains a widely used and clinically interpretable measure of lumbar-related disability, which facilitated comparison with previous studies.

Fifth, no formal intra-rater or inter-rater reliability analysis was performed for the McGill or FMS assessments, and this may have introduced measurement error, particularly in the more subjective FMS components. Sixth, no a priori sample size calculation was performed. Finally, potentially relevant confounders such as training load, type of sport, previous injury history, and psychosocial factors were not included and may partly explain the weak associations observed. In addition, the selected measures reflect trunk endurance and movement performance rather than the full multidimensional construct of motor control, so this term should be interpreted cautiously in the present context.

In contrast, the McGill and FMS are useful and standardized tests; however, they indirectly reflect the construct of motor control and may be influenced by mobility, stabilization strategies, or scoring criteria. Robust methods were used (non-parametric tests and FDR adjustment); however the multivariable analysis was adjusted for only basic covariates; therefore, potentially relevant confounders such as training load, type of sport, previous injury history, and psychosocial factors were not included and may partly explain the weak associations observed [34,35].

For these reasons, the present findings should be interpreted as descriptive of concurrent associations in a specific, low-disability physically active cohort. They do not support risk prediction, causal inference, or direct clinical application beyond this physically active young adult cohort.

## 5. Conclusions

In physically active young adults with very low disability levels, trunk endurance tests and selected movement-pattern measures provided complementary descriptive information on current lumbar-related function. However, the observed associations with ODI were modest, the explanatory capacity of the multivariable model was low, and the finding related to Inline Lunge asymmetry should be interpreted as exploratory because of the very small subgroup size. Accordingly, these results should be understood as population-specific and hypothesis-generating rather than predictive. Their main contribution is to describe concurrent associations in a low-disability active cohort and to help guide future studies in broader and more clinically relevant populations.

## Figures and Tables

**Table 1:** Participant characteristics and descriptive statistics.

Characteristic	Mean ± SD	Median (IQR)	Range
Age (years)	22.85 ± 2.77	22.00 (IQR 3.00)	19.00 to 31.00
Weight (kg)	68.06 ± 11.11	68.60 (IQR 12.00)	46.00 to 110.00
Height (m)	1.75 ± 0.09	1.76 (IQR 0.11)	1.56 to 1.94
Body mass index (kg/m^2^)	22.20 ± 2.68	22.01 (IQR 3.57)	16.81 to 33.95
Oswestry Disability Index total	4.00 ± 5.69	2.00 (IQR 5.00)	0.00 to 26.00
McGill prone plank (s)	133.28 ± 38.32	126.00 (IQR 30.00)	49.00 to 240.00
McGill trunk flexor endurance (s)	124.24 ± 68.49	118.00 (IQR 71.50)	27.00 to 420.00
McGill side bridge right (s)	76.75 ± 34.52	70.00 (IQR 36.00)	10.00 to 200.00
McGill side bridge left (s)	78.27 ± 35.53	70.00 (IQR 33.00)	13.00 to 200.00
McGill sum of indices	2.13 ± 0.25	2.14 (IQR 0.24)	1.61 to 2.58
FMS total score	9.71 ± 1.57	10.00 (IQR 2.00)	3.50 to 12.00

Note: Continuous variables are presented as mean (SD), median (IQR), and range. Categorical variables are presented as numbers (*n*) and percentages (%).

**Table 2:** Sex comparisons for key outcomes.

Outcome	Female (*n*, Mean ± SD)	Male (*n*, Mean ± SD)	W	*p*-Value
Age (years)	15, 21.20 ± 1.86	56, 23.29 ± 2.81	216	0.004
Body mass index (kg/m^2^)	15, 21.49 ± 2.67	56, 22.40 ± 2.68	334	0.228
Oswestry Disability Index total	15, 3.73 ± 4.13	56, 4.07 ± 6.07	440	0.776
FMS total score	15, 8.97 ± 2.08	56, 9.91 ± 1.35	303.5	0.096

Note: Group differences were assessed using the Wilcoxon rank-sum test.

**Table 3:** Associations between FMS asymmetry flags and outcomes.

Asymmetry	Outcome	No (*n*, Mean ± SD)	Yes (*n*, Mean ± SD)	W	*p*-Value	*p*-Value (FDR)
FMS Inline Lunge asymmetry	Oswestry Disability Index total	67, 3.07 ± 3.56	4, 19.50 ± 11.70	34.5	0.011	0.042
FMS Inline Lunge asymmetry	FMS total score	67, 9.81 ± 1.50	4, 8.12 ± 1.97	211	0.052	0.103
FMS Rotary Stability asymmetry	Oswestry Disability Index total	60, 3.70 ± 5.14	11, 5.64 ± 8.19	314.5	0.805	0.808
FMS Rotary Stability asymmetry	FMS total score	60, 9.78 ± 1.42	11, 9.36 ± 2.27	345.5	0.808	0.808

Note: Group differences were assessed using the Wilcoxon rank-sum test. *p*-values were adjusted for multiple comparisons using the false discovery rate (FDR).

**Table 4:** Top Spearman correlations across ODI, McGill endurance tests, and FMS outcomes.

Variable 1	Variable 2	Spearman ρ	*p*-Value	*p*-Value (FDR)
McGill side bridge right (s)	McGill side bridge left (s)	0.897	<0.001	<0.001
FMS total score	FMS Trunk Stability Push-up	0.626	<0.001	<0.001
McGill prone plank (s)	McGill trunk flexor endurance (s)	0.553	<0.001	<0.001
McGill trunk flexor endurance (s)	McGill side bridge left (s)	0.519	<0.001	<0.001
FMS total score	FMS Deep Squat	0.48	<0.001	<0.001
McGill prone plank (s)	McGill side bridge left (s)	0.472	<0.001	<0.001
FMS Trunk Stability Push-up	McGill side bridge left (s)	0.465	<0.001	<0.001
FMS Trunk Stability Push-up	McGill side bridge right (s)	0.458	<0.001	<0.001
McGill trunk flexor endurance (s)	McGill side bridge right (s)	0.446	<0.001	<0.001
McGill prone plank (s)	McGill side bridge right (s)	0.428	<0.001	0.001

Note: Spearman correlation coefficients are shown with FDR-adjusted *p*-values.

**Table 5:** Multivariable linear regression model predicting total ODI.

Predictor	β (SE)	t	*p*-Value
Intercept	7.342 (9.204)	0.798	0.428
FMS total score	−0.833 (0.455)	−1.833	0.071
Age (years)	0.135 (0.261)	0.517	0.607
Body mass index (kg/m^2^)	0.047 (0.260)	0.18	0.858
Sex (Male vs. Female)	0.801 (1.817)	0.441	0.661

Note: The model included the FMS total score, age, BMI, and sex.

## Data Availability

The datasets used and analyzed during the current study are available from the corresponding author upon reasonable request owing to privacy and ethical restrictions.

## Abbreviations

The following abbreviations are used in this manuscript:

LBP	Low back pain
FMS	Functional Movement Screen
ODI	Oswestry Disability Index
FDR	False Discovery Rate
OLS	Ordinary Least Squares
